# Association of Frailty and Postoperative Complications With Unplanned Readmissions After Elective Outpatient Surgery

**DOI:** 10.1001/jamanetworkopen.2019.4330

**Published:** 2019-05-24

**Authors:** Kara A. Rothenberg, Jordan R. Stern, Elizabeth L. George, Amber W. Trickey, Arden M. Morris, Daniel E. Hall, Jason M. Johanning, Mary T. Hawn, Shipra Arya

**Affiliations:** 1 Stanford–Surgery Policy Improvement Research and Education Center, Department of Surgery, Stanford University School of Medicine, Stanford, California; 2 Surgical Service, Veterans Affairs Palo Alto Health System, Palo Alto, California; 3 Department of Surgery, University of Pittsburgh School of Medicine, Pittsburgh, Pennsylvania; 4 Center for Health Equity Research and Promotion, Veterans Affairs Pittsburgh Healthcare System, Pittsburgh, Pennsylvania; 5 Wolffe Center at UPMC, University of Pittsburgh Medical Center, Pittsburgh, Pennsylvania; 6 Department of Surgery, University of Nebraska College of Medicine, Omaha

## Abstract

**Importance:**

Ambulatory surgery in geriatric populations is increasingly prevalent. Prior studies have demonstrated the association between frailty and readmissions in the inpatient setting. However, few data exist regarding the association between frailty and readmissions after outpatient procedures.

**Objective:**

To examine the association between frailty and 30-day unplanned readmissions after elective outpatient surgical procedures as well as the potential mediation of surgical complications.

**Design, Setting, and Participants:**

In this retrospective cohort study of elective outpatient procedures from 2012 and 2013 in the National Surgical Quality Improvement Program (NSQIP) database, 417 840 patients who underwent elective outpatient procedures were stratified into cohorts of individuals with a length of stay (LOS) of 0 days (LOS = 0) and those with a LOS of 1 or more days (LOS ≥ 1). Statistical analysis was performed from June 1, 2018, to March 31, 2019.

**Exposure:**

Frailty, as measured by the Risk Analysis Index.

**Main Outcomes and Measures:**

The main outcome was 30-day unplanned readmission.

**Results:**

Of the 417 840 patients in this study, 59.2% were women and unplanned readmission occurred in 2.3% of the cohort overall (LOS = 0, 2.0%; LOS ≥ 1, 3.4%). Frail patients (mean [SD] age, 64.9 [15.5] years) were more likely than nonfrail patients (mean [SD] age, 35.0 [15.8] years) to have an unplanned readmission in both LOS cohorts (LOS = 0, 8.3% vs 1.9%; LOS ≥ 1, 8.5% vs 3.2%; *P* < .001). Frail patients were also more likely than nonfrail patients to experience complications in both cohorts (LOS = 0, 6.9% vs 2.5%; LOS ≥ 1, 9.8% vs 4.6%; *P* < .001). In multivariate analysis, frailty doubled the risk of unplanned readmission (LOS = 0: adjusted relative risk [RR], 2.1; 95% CI, 2.0-2.3; LOS ≥ 1: adjusted RR, 1.8; 95% CI, 1.6-2.1). Complications occurred in 3.1% of the entire cohort, and frailty was associated with increased risk of complications (unadjusted RR, 2.6; 95% CI, 2.4-2.8). Mediation analysis confirmed that complications are a significant mediator in the association between frailty and readmissions; however, it also indicated that the association of frailty with readmission was only partially mediated by complications (LOS = 0, 22.8%; LOS ≥ 1, 29.3%).

**Conclusions and Relevance:**

These findings suggest that frailty is a significant risk factor for unplanned readmission after elective outpatient surgery both independently and when partially mediated through increased complications. Screening for frailty might inform the development of interventions to decrease unplanned readmissions, including those for outpatient procedures.

## Key Points

**Question:**

Is frailty, as measured by the Risk Analysis Index, associated with risk for unplanned readmission after elective outpatient surgery?

**Findings:**

In this cohort study of 417 840 patients in the National Surgical Quality Improvement Program, frailty was associated with an increased risk for unplanned readmissions after elective outpatient surgery. Mediation analysis showed that the association of frailty with readmission was partially mediated by occurrence of complications.

**Meaning:**

Surgical patients may benefit from screening for frailty to decrease complications and unplanned readmissions after surgery.

## Teaser

This cohort study uses National Surgical Quality Improvement Program (NSQIP) data to examine the association between frailty and 30-day unplanned readmissions after elective outpatient surgical procedures.

## Introduction

Unplanned hospital readmission represents a significant financial burden to the medical system and is closely tied to postoperative complications.^1,2,3^ Most studies on readmission thus far have focused on inpatient surgical procedures.^4,5,6,7^ Less is known about readmissions for outpatient procedures or those in the ambulatory setting despite an increased frequency of such procedures nationwide.^8^ The cost savings associated with ambulatory surgery centers has led to increased use of ambulatory surgery by the elderly for common procedures.^9^ Generally, outpatient surgery has been difficult to study because of its inconsistent definition in databases and the literature.^10,11,12^ In addition, Medicare beneficiaries are increasingly being hospitalized under “observation” status despite staying in the hospital up to 3 days.^13^ Of the few studies that have focused on readmissions in the outpatient surgery setting, readmissions have been linked to surgical complications, obesity, and increased patient age.^14,15^

Frailty is a syndrome of increased vulnerability to a stressor, which can disrupt physiological homeostasis and degrade health status.^16,17^ Frail patients have an increased risk of postoperative complications in many clinical contexts.^18,19,20^ More recently, however, research has focused on the association of frailty with quality metrics, such as failure to rescue (ie, death after a potentially preventable complication) and hospital readmissions,^21,22,23,24,25^ both of which are more likely to occur in frail individuals. The Risk Analysis Index (RAI) is a validated frailty assessment tool that is able to estimate 30-, 180-, and 365-day postoperative mortality.^26,27^ Its association with readmission risk has not been studied, to our knowledge.^28^

In this study, we used the American College of Surgeons National Surgical Quality Improvement Program (NSQIP) database to identify patients undergoing elective outpatient surgery. The objective was to determine the association of frailty with the risk of 30-day unplanned readmission (as defined by the NSQIP). We also explored the potential mediation of complications on the association between frailty and unplanned readmissions. We hypothesized that frail patients would have higher rates of unplanned readmissions in the outpatient setting and that this association would be mediated by the occurrence of postoperative complications.

## Methods

### Cohort Creation

We used the NSQIP participant use file databases from 2012^29^ and 2013^30^ to retrospectively identify patients who underwent outpatient elective surgery. The NSQIP includes 2 variables indicative of ambulatory outpatient surgery: INOUT (“the hospital’s definition of inpatient and outpatient status”) and ELECTSURG (“if the patient is brought to the hospital or facility for a scheduled [elective] surgery from their home or normal living situation on the day that the procedure is performed”). We defined elective surgery as cases coded as both outpatient (INOUT = outpatient) and elective (ELECTSURG = yes). All other cases were considered inpatient or nonelective procedures. Patients whose physical status was classified as American Society of Anesthesiologists classification V were excluded from the study (n = 21), as this classification did not seem to be appropriate for patients undergoing outpatient elective surgery. This study was reviewed by the Stanford University Institutional Review Board and the need for informed consent from study participants was waived because the data were deidentified. The reporting of this study conforms to the Strengthening the Reporting of Observational Studies in Epidemiology (STROBE) reporting guideline.^31^

Within the NSQIP, all patients are considered “admitted” to the operating room for the index procedure. Some of these patients are discharged home within 24 hours as traditional ambulatory surgery (ie, length of stay of 0 days [LOS = 0]), whereas others—23.2% of our cohort—remain admitted for longer (LOS of 1 or more days [LOS ≥ 1]). Because we did not have information on the reasons for admission or observation (considered the same event in the NSQIP) after outpatient surgery, and we observed significant differences in age and comorbidity burden as well as frailty in the 2 LOS groups (eTable 1 in the Supplement), the cohort was stratified for all subsequent analyses into LOS = 0 and LOS ≥ 1.

### Exposure: Frailty

Frailty was assessed using the RAI.^26^ The RAI score was determined by the following NSQIP variables: sex, age, disseminated cancer, weight loss, renal failure, congestive heart failure, dyspnea, transfer status, functional status, and cognitive status (impaired sensorium, coma, or prior stroke); full RAI weighted scoring is described in eTable 2 in the Supplement. Patients with incomplete information to assess frailty or LOS were excluded (0.9% of cohort; eFigure in the Supplement). Frailty status was dichotomized, with RAI scores of 30 or more defining a frail patient based on recent work validating this recalibrated version of the RAI.^27^ Complications were grouped into major (deep space infection or organ space infection, wound disruption, pneumonia, unplanned intubation, pulmonary embolism, prolonged ventilator use, acute renal failure, stroke, cardiac arrest, myocardial infarction, sepsis or septic shock, and return to operating room) and minor (superficial surgical site infection, deep venous thrombosis or thromboembolism [considered the same event in the NSQIP], progressive renal insufficiency, urinary tract infection, and blood transfusion) categories.^21^

### Outcome: Unplanned Readmission

Our outcome variable was UNPLANREADMISSION,^29^ defined by the NSQIP registry as “any readmission (to the same or another hospital) for a postoperative occurrence likely related to the principal surgical procedure within 30 days of the procedure.” To maintain consistent terminology with the NSQIP registry, we therefore chose to refer to these as “readmissions,” regardless of index LOS. Thus, the initial stay for every patient in this study regardless of LOS was considered an “admission.” Because patients who died during or immediately after surgery would therefore not be at risk for our outcome of interest, by definition, we further excluded any patients who died during the initial surgery admission (n = 54; eFigure in the Supplement).

### Statistical Analysis

Statistical analysis was performed from June 1, 2018, to March 31, 2019. Univariate χ^2^ tests, Fisher exact tests, and independent-sample *t* tests were performed to compare patient baseline characteristics by frailty status. Multivariable log-binomial regression models^32^ were calculated to investigate associations between frailty, postoperative complications, and readmission, with adjustment for demographic and clinical characteristics, including American Society of Anesthesiologists Classification, preoperative hematocrit, smoking, and comorbidities not included in the RAI. Because readmission was likely associated with postoperative complications, 2 multivariable logistic regression models were created to evaluate the frailty-specific relative risk (RR) for unplanned readmissions. One model consisted of preoperative factors only, including frailty. The other contained both preoperative risk factors and postoperative complications. Because complications occurred in only 3.1% of the cohort, a post hoc multivariate model was also created to evaluate the association between frailty and readmissions for the 405 029 patients who did not experience any complications.

We evaluated the association of frailty with unplanned readmissions as mediated by postoperative complications because complications may exist in the causal pathway between frailty and readmissions (ie, frailty may lead to a complication, which may cause an unplanned readmission). We determined the degree to which the occurrence of any postoperative complication influenced the association between frailty and unplanned 30-day readmission.^33^ Three multivariable logistic regression models were calculated to carry out the product method with standardized effects. Partial mediation was evaluated by determining the association of frailty with complications for both LOS cohorts and the association of complications with readmissions, adjusted for frailty. Using these estimates, the indirect effect and proportion mediated were calculated (eAppendix in the Supplement).^34,35,36^

All *P* values were from 2-sided tests, and results were deemed statistically significant at *P* < .05. All statistical analyses were performed using Stata, version 15 (StataCorp LP).

## Results

The final cohort consisted of 417 840 patients undergoing elective outpatient surgical procedures with complete RAI data (eFigure in the Supplement), of whom 59.2% were women, 40.8% were men, and the mean (SD) age was 36.3 (16.3) years. General surgery procedures were the most common outpatient procedures (cholecystectomy and hernia procedures) in both frail patients (38.1%) and nonfrail patients (39.5%) (eTable 3 in the Supplement). After general surgery, breast and arthroscopic procedures were the most common in nonfrail patients (32.6%), whereas urologic and skin and soft-tissue procedures were more common in frail patients (36.2%). Patients with longer index LOS were significantly older (mean [SD] age, 38.1 [15.8] vs 35.8 [16.4] years; *P* < .001), had a higher prevalence of frailty (3.1% vs 2.5%; *P* < .001), and had a higher comorbidity burden (eTable 1 in the Supplement). Using an RAI score of 30 or more to define frailty, 8079 of 321 104 patients (2.5%) were classified as frail in the LOS = 0 group, and 3041 of 96 736 patients (3.1%) were classified as frail in the LOS ≥ 1 group (Table 1). As expected, frail patients were significantly older than nonfrail patients (LOS = 0: mean [SD] age, 64.9 [15.5] vs 35.0 [15.8] years; LOS ≥ 1: mean [SD] age, 63.2 [17.5] vs 37.2 [15.0] years; *P* < .001). In both LOS cohorts, frail patients had higher rates than nonfrail patients of a variety of comorbidities including insulin-dependent diabetes (LOS = 0, 12.5% vs 3.2%; *P* < .001; LOS ≥ 1, 10.5% vs 4.3%; *P* < .001), non–insulin-dependent diabetes (LOS = 0, 11.8% vs 7.1%; *P* < .001; LOS ≥ 1, 12.2% vs 9.1%; *P* < .001), hypertension (LOS = 0, 70.2% vs 34.4%; *P* < .001; LOS ≥ 1, 60.0% vs 42.2%; *P* < .001), and congestive heart failure (LOS = 0, 3.9% vs 0.1%; *P* < .001; LOS ≥ 1, 3.9% vs 0.1%; *P* < .001).

**Table 1.** Patient Demographics

Characteristic	LOS = 0 (n = 321 104 [76.8%])			LOS ≥ 1 (n = 96 736 [23.2%])		
	Nonfrail (n = 313 025 [97.5%])	Frail (n = 8079 [2.5%])	*P* Value	Nonfrail (n = 93 695 [96.9%])	Frail (n = 3041 [3.1%])	*P* Value
Age, mean (SD), y	35.0 (15.8)	64.9 (15.5)	<.001	37.2 (15.0)	63.2 (17.5)	<.001
Female sex, No. (%)	176 083 (56.3)	2527 (31.3)	<.001	67 607 (72.2)	1290 (42.4)	<.001
Diabetes, No. (%)						
Insulin dependent	9897 (3.2)	1012 (12.5)	<.001	4049 (4.3)	318 (10.5)	<.001
Non–insulin dependent	22 271 (7.1)	950 (11.8)		8551 (9.1)	370 (12.2)	
Hypertension, No. (%)	107 807 (34.4)	5673 (70.2)	<.001	39 535 (42.2)	2099 (69.0)	<.001
Congestive heart failure, No. (%)	287 (0.1)	315 (3.9)	<.001	129 (0.1)	119 (3.9)	<.001
Coronary artery disease, No. (%)	1522 (0.5)	156 (1.9)	<.001	551 (0.6)	77 (2.5)	<.001
Disseminated cancer, No. (%)	8 (<0.01)	1350 (16.7)	<.001	11 (0.01)	792 (26.0)	<.001
COPD, No. (%)	7138 (2.3)	751 (9.3)	<.001	3014 (3.2)	278 (9.2)	<.001
Current smoker, No. (%)	54 262 (17.3)	642 (8.0)	<.001	16 175 (17.3)	239 (7.9)	<.001
Preoperative hematocrit, No. (%)						
Q1: 43.2%-60.0%	57 718 (18.4)	875 (10.8)	<.001	16 572 (17.7)	332 (10.9)	<.001
Q2: 40.6%-43.1%	52 084 (16.6)	1015 (12.6)		18 904 (20.2)	471 (15.5)	
Q3: 37.9%-40.5%	54 860 (17.5)	1470 (18.2)		22 402 (23.9)	623 (20.5)	
Q4: 8.0%-37.8%	47 995 (15.3)	3604 (44.6)		21 569 (23.0)	1360 (44.7)	
Unknown or missing	100 368 (32.1)	1115 (13.8)		14 248 (15.2)	255 (8.4)	
ASA Classification, No. (%)						
I: No disturbance	55 693 (17.8)	66 (0.8)	<.001	7301 (7.8)	18 (0.6)	<.001
II: Mild disturbance	178 818 (57.1)	1704 (21.1)		51 917 (55.4)	635 (20.9)	
III: Severe disturbance	73 203 (23.4)	5016 (62.1)		32 429 (34.6)	1990 (65.4)	
IV: Life threatening	3593 (1.2)	1171 (14.5)		1801 (1.9)	377 (12.4)	
Unknown or missing	1718 (0.6)	122 (1.5)		247 (0.3)	21 (0.7)	
Corticosteroid use, No. (%)	6252 (2.0)	445 (5.5)	<.001	2445 (2.6)	190 (6.3)	<.001
Bleeding disorder, No. (%)	5337 (1.7)	8079 (9.2)	<.001	2227 (2.4)	314 (10.3)	<.001

Abbreviations: ASA, American Society of Anesthesiologists; COPD, chronic obstructive pulmonary disease; LOS, length of stay in days; Q, quartile.

The 30-day complication rates by frailty status within each LOS cohort are outlined in Table 2. Overall, postoperative complications were infrequent, with only 2.6% of patients in the LOS = 0 cohort experiencing complications and 4.8% of patients in the LOS ≥ 1 cohort experiencing complications. However, complications were more frequent in frail patients than in nonfrail patients (LOS = 0, 6.9% vs 2.5%; *P* < .001; LOS ≥ 1, 9.8% vs 4.6%; *P* < .001). Regarding minor complications, frail patients in both cohorts were more likely than nonfrail patients to experience deep venous thrombosis (LOS = 0, 0.2% vs 0.2%; *P* = .003; LOS ≥ 1, 0.4% vs 0.1%; *P* < .001), renal insufficiency (LOS = 0, 0.2% vs 0.02%; *P* < .001; LOS ≥ 1, 0.3% vs 0.03%; *P* < .001), urinary tract infections (LOS = 0, 1.6% vs 0.4%; *P* < .001; LOS ≥ 1, 2.2% vs 0.9%; *P* < .001), and bleeding requiring transfusion (LOS = 0, 0.4% vs 0.04%; *P* < .001; LOS ≥ 1, 2.3% vs 0.6%; *P* < .001). Major complications including respiratory, cardiac, and infectious processes were also more common in frail patients, particularly in the LOS = 0 group. In the LOS ≥ 1 group, rates of wound infections, wound disruptions, airway complications, and cardiac arrest did not differ significantly between frail and nonfrail patients.

**Table 2.** Complications Within 30 Days

Complication	LOS = 0 (n = 321 104 [76.8%])			LOS ≥ 1 (n = 96 736 [23.2%])		
	Nonfrail (n = 313 025 [97.5%])	Frail (n = 8079 [2.5%])	*P* Value	Nonfrail (n = 93 695 [96.9%])	Frail (n = 3041 [3.1%])	*P* Value
Minor, No. (%)						
Superficial surgical site infection	2025 (0.7)	43 (0.5)	.20	763 (0.8)	34 (1.1)	.07
DVT or thromboembolism	349 (0.1)	18 (0.2)	.003	132 (0.1)	13 (0.4)	<.001
Renal insufficiency	61 (0.02)	14 (0.2)	<.001	29 (0.03)	8 (0.3)	<.001
Urinary tract infection	1378 (0.4)	131 (1.6)	<.001	819 (0.9)	68 (2.2)	<.001
Bleeding requiring transfusion	133 (0.04)	28 (0.4)	<.001	575 (0.6)	69 (2.3)	<.001
Any minor complication	3896 (1.2)	230 (2.9)	<.001	2265 (2.4)	181 (6.0)	<.001
Major, No. (%)						
SSI						
Deep incisional	440 (0.1)	22 (0.3)	.002	211 (0.2)	4 (0.1)	.43
Organ space	376 (0.1)	13 (0.2)	.30	314 (0.3)	15 (0.5)	.14
Wound disruption	283 (0.09)	14 (0.2)	.02	175 (0.2)	10 (0.3)	.08
Pneumonia	213 (0.07)	50 (0.6)	<.001	136 (0.2)	21 (0.7)	<.001
Unplanned intubation	91 (0.03)	25 (0.3)	<.001	94 (0.1)	5 (0.2)	.24
Pulmonary embolism	181 (0.06)	10 (0.1)	.02	107 (0.1)	10 (0.3)	.001
Ventilator >48 h	37 (0.01)	4 (0.05)	.02	45 (0.05)	4 (0.1)	.07
Acute renal failure	46 (0.01)	10 (0.1)	<.001	29 (0.03)	5 (0.2)	.004
Stroke or CVA	43 (0.01)	20 (0.3)	<.001	34 (0.04)	6 (0.2)	<.001
Cardiac arrest requiring CPR	37 (0.01)	12 (0.2)	<.001	19 (0.02)	1 (0.03)	.47
Myocardial infarction	87 (0.03)	20 (0.3)	<.001	43 (0.05)	11 (0.4)	<.001
Sepsis	351 (0.1)	47 (0.6)	<.001	216 (0.2)	22 (0.7)	<.001
Septic shock	62 (0.02)	26 (0.3)	<.001	46 (0.05)	7 (0.2)	<.001
Return to operating room	2908 (0.9)	213 (2.6)	<.001	1560 (1.7)	88 (2.9)	<.001
Any major complication	4242 (1.4)	381 (4.7)	<.001	2402 (2.6)	155 (5.1)	<.001
Any complication, No. (%)	7659 (2.5)	555 (6.9)	<.001	4300 (4.6)	297 (9.8)	<.001
Unplanned readmission, No. (%)	5814 (1.9)	669 (8.3)	<.001	3018 (3.2)	258 (8.5)	<.001

Abbreviations: CPR, cardiopulmonary resuscitation; CVA, cerebrovascular accident; DVT, deep venous thrombosis; LOS, length of stay in days; SSI, surgical site infection.

**Figure 1.**
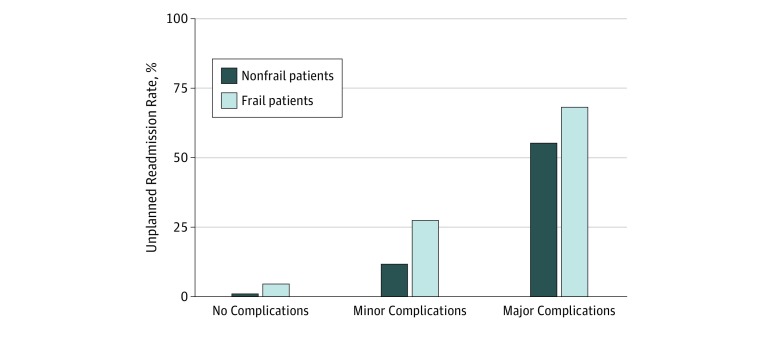
Thirty-Day Unplanned Readmissions by Frailty and Complication Status

Overall, 30-day unplanned readmission occurred in 2.3% of the total cohort (LOS = 0, 2.0%; LOS ≥ 1, 3.4%). Patients with minor and major complications comprised most unplanned readmissions (Figure 1), and frail patients had more unplanned readmissions than nonfrail patients in both cohorts (LOS = 0, 8.3% vs 1.9%; LOS ≥ 1, 8.5% vs 3.2%; *P* < .001), whether they had no complication, a minor complication, or a major complication. Complications occurred in 3.1% of the entire cohort, and frailty was associated with increased risk of complications (unadjusted RR, 2.6; 95% CI, 2.4-2.8). Both frailty status and occurrence of complications were associated with readmissions. Table 3 shows the 2 multivariate logistic regression models examining the association of frailty with readmissions for each LOS cohort. In the model with only preoperative factors, the RR for readmission was significantly higher for frail patients (LOS = 0: RR 2.1; 95% CI, 2.0-2.3; LOS ≥ 1: RR, 1.8; 95% CI, 1.6-2.1) after adjustment for covariates. Although minor and major complications increased the RR significantly, the association of frailty with readmission was attenuated (LOS = 0: RR, 1.5; 95% CI, 1.3-1.6; LOS ≥ 1: RR, 1.5; 95% CI, 1.3-1.7) after adjustment for complications and other covariates.

**Table 3.** Regression Models Demonstrating the Adjusted Relative Risk for Unplanned Readmission

Model	Adjusted Relative Risk (95% CI)	
	LOS = 0 (n = 321 104 [76.8%])	LOS ≥ 1 (n = 96 736 [23.2%])
**With Preoperative Factors Only**		
Frailty	2.1 (2.0-2.3)	1.8 (1.6-2.1)
Diabetes [reference: none]		
Insulin dependent	1.6 (1.4-1.7)	1.3 (1.1-1.4)
Non–insulin dependent	1.0 (1.0-1.1)	1.0 (0.9-1.1)
Hypertension	1.1 (1.1-1.2)	1.1 (1.0-1.2)
Coronary artery disease	1.1 (0.9-1.4)	1.0 (0.7-1.4)
COPD	1.5 (1.4-1.7)	1.3 (1.1-1.5)
Current smoker	1.2 (1.1-1.3)	1.3 (1.1-1.4)
Hematocrit quartiles [reference: 1: 43.2%-60.0%}		
2: 40.6%-43.1%	1.1 (1.0-1.2)	1.0 (0.9-1.1)
3: 37.9%-40.5%	1.2 (1.1-1.3)	1.0 (0.9-1.1)
4: 8.0%-37.8%	1.7 (1.6-1.9)	1.2 (1.1-1.4)
Missing	0.9 (0.8-1.0)	0.9 (0.8-1.0)
ASA classification [reference: I]		
II	1.5 (1.3-1.6)	1.3 (1.1-1.5)
III	2.4 (2.2-2.7)	1.9 (1.6-2.3)
IV	3.4 (2.9-4.0)	2.9 (2.3-3.7)
Missing	1.8 (1.3-2.5)	2.6 (1.6-4.2)
Corticosteroid use	1.8 (1.6-2.0)	1.5 (1.3-1.8)
Bleeding disorder	1.6 (1.4-1.8)	1.4 (1.2-1.6)
**With Preoperative and Postoperative Factors **		
Frailty	1.5 (1.3-1.6)	1.5 (1.3-1.7)
Complication [reference: none]		
Minor	10.8 (9.8-11.9)	6.0 (5.3-6.9)
Major	42.2 (40.0-44.5)	27.0 (25.1-29.1)
Diabetes [reference: none]		
Type 1	1.1 (1.0-1.2)	1.1 (1.0-1.3)
Type 2	1.0 (1.0-1.1)	1.0 (0.9-1.1)
Hypertension	1.1 (1.0-1.2)	1.1 (1.0-1.2)
Coronary artery disease	1.1 (0.9-1.4)	1.0 (0.7-1.4)
COPD	1.3 (1.2-1.4)	1.2 (1.0-1.3)
Current smoker	1.1 (1.0-1.2)	1.1 (1.0-1.2)
Hematocrit quartiles [reference: 1: 43.2%-60.0%]		
2: 40.6%-43.1%	1.0 (1.0-1.1)	1.2 (1.0-1.5)
3: 37.9%-40.5%	1.1 (1.0-1.2)	1.6 (1.4-2.0)
4: 8.0%-37.8%	1.4 (1.3-1.5)	1.9 (1.5-2.4)
Missing	0.9 (0.8-0.9)	2.0 (1.2-3.2)
ASA classification [reference: I]		
II	1.4 (1.2-1.5)	1.2 (1.0-1.5)
III	1.9 (1.7-2.1)	1.6 (1.4-2.0)
IV	2.2 (1.9-2.5)	1.9 (1.5-2.4)
Missing	1.4 (1.0-1.9)	2.0 (1.2-3.2)
Corticosteroid use	1.5 (1.3-1.7)	1.3 (1.1-1.5)
Bleeding disorder	1.2 (1.1-1.4)	1.2 (1.0-1.4)

Abbreviations: ASA, American Society of Anesthesiologists; COPD, chronic obstructive pulmonary disease; LOS, length of stay in days.

**Figure 2.**
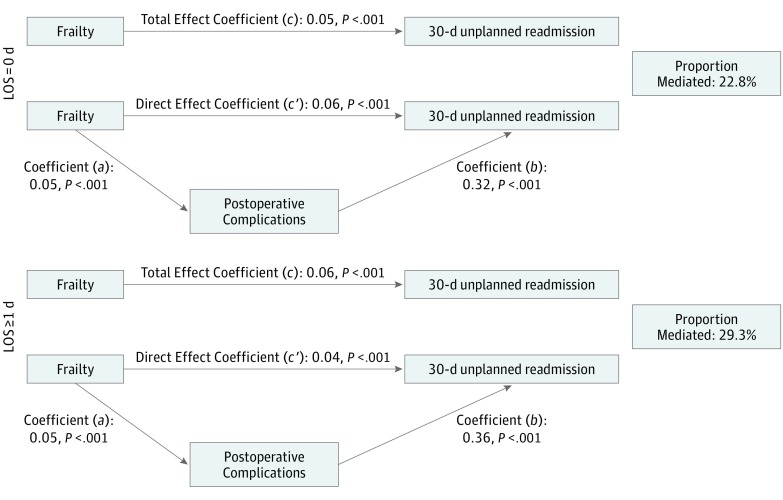
Mediation Analysis LOS indicates length of stay.

In our post hoc multivariate model with patients who did not experience any complications, frailty was associated with double the risk of readmission (LOS = 0: RR, 2.2; 95% CI, 1.9-2.5; LOS ≥ 1: RR, 1.8; 95% CI, 1.5-2.2). In both cohorts, mediation analysis showed a significant association between frailty and unplanned readmission (Figure 2; coefficient *c*) and frailty and postoperative complications (Figure 2; coefficient *a*). Complications were significantly associated with readmissions (Figure 2; coefficient *b*) and when adjusted for frailty (Figure 2; coefficient *c*′) for both LOS cohorts. The overall mediation analysis demonstrated that the indirect (mediation) association of frailty with readmissions through the occurrence of any complications was significant (Figure 2). The estimated proportions of associations between frailty and readmissions mediated by complications were 22.8% for the LOS = 0 group and 29.3% for the LOS ≥ 1 group.

We also examined reasons for readmissions and found that 1985 of 7774 (25.5%) were missing a readmission reason (eTable 4 in the Supplement). Of the patients with a documented NSQIP-prespecified complication, most complications (70.9%) were categorized as other. To better characterize these reasons for readmission, we recategorized the NSQIP complications per Merkow et al.^3^ With this categorization, surgical site infections (nonfrail, 18.6%; frail, 7.5%; *P* = .01), gastrointestinal (eg, ileus and emesis) complications (nonfrail, 16.4%; frail, 10.1%; *P* < .001), and bleeding (nonfrail, 11.4%; frail, 10.5%; *P* < .001) were the most common reasons for readmission. Frail patients were significantly more likely than nonfrail patients to be hospitalized for renal or genitourinary complications (5.6% vs 3.7%; *P* < .001), neurologic complications (3.3% vs 1.5%; *P* < .001), cancer (6.2% vs 5.0%; *P* < .001), cardiac complications (8.8% vs 3.1%; *P* < .001), orthopedic complications (2.3% vs 2.0%; *P* < .001), other medical complications (6.9% vs 5.6%; *P* < .001), pulmonary complications (9.4% vs 4.4%; *P* < .001), sepsis (7.1% vs 2.8%; *P* < .001), and vascular complications (2.3% vs 1.2%; *P* < .001).

## Discussion

Our study examines in detail the associations of frailty with unplanned readmissions after elective outpatient surgical procedures and the mediation of the risk of readmission that occurs through increased complications in frail patients. We found that frailty doubled the risk of unplanned readmission in the overall cohort. The rate of complications was nearly 3-fold higher in frail patients in both LOS cohorts, and frailty was significantly associated with unplanned readmission, as were postoperative complications in multivariate analysis. The association of frailty with unplanned readmission was partially mediated (LOS = 0, 22.8%; LOS ≥ 1, 29.3%) through complications.

The overall unplanned readmission rate in our study (2.3%) is comparable with prior studies examining unplanned readmission after various outpatient procedures.^14,37,38,39^ In a retrospective analysis using the 2012 NSQIP data set, De Oliveira et al^15^ demonstrated a significant association between advanced age (≥70 years) and unplanned readmission and similarly found that a large proportion of readmissions were associated with postoperative complications. Although age is a component of the RAI, frailty is not defined by age or comorbidities alone. The RAI includes only renal failure and congestive heart failure as 2 comorbidities while incorporating other important deficits, encompassing the physical, functional, nutritional, social, and cognitive domains of frailty. As shown in eTable 2 in the Supplement, each of these domains contributes significant weight to the overall score; for example, functional status contributes up to 16 points to the overall RAI (range, 0-81). Thus, frailty as measured by the RAI has been shown to be a more accurate representation of patients’ physiological reserve and ability to survive adverse surgical events.^20^ A recent, large NSQIP study^40^ evaluated more than 140 000 patients undergoing ambulatory general surgery procedures to determine the predictive value of the Modified Frailty Index on postoperative complications. This group showed that an increasing Modified Frailty Index was associated with a higher risk of complications, as high as 4-fold in the group with the highest level of frailty. Our study echoes these results and further shows that the complications that occur in frail patients can lead to increased unplanned readmission after outpatient surgical procedures.

Increased risk of readmission in frail patients has been more consistently shown after inpatient surgical procedures. Frail patients (as measured by the Modified Frailty Index) are more likely to be readmitted after inpatient orthopedic, general, or vascular surgery,^25,41,42^ although the association seen was significantly less pronounced than in our study, with an odds ratio of only 1.11. Although complications were more frequent among frail patients, the authors did not specifically address the contribution of complications to readmission. The association between postoperative complications and readmission has been well described,^23,43,44^ and our analysis confirms that readmissions among frail patients are mediated by the occurrence of complications. These findings add to existing work using the RAI,^20,27,45^ highlighting the RAI’s utility as a preoperative screening tool for quality improvement work, even in the case of elective outpatient surgery. However, the RAI’s utility as a preoperative screening tool does not undermine the role of recognition and early treatment for complications.

Merkow et al^3^ analyzed the 2012 NSQIP data for all procedures and 6 selected procedures for reasons of readmissions and found surgical site infections, gastrointestinal complications (ileus or obstruction), and bleeding as the most common causes of readmission. Similarly, our analysis of outpatient surgical procedures shows surgical site infections, bleeding, and gastrointestinal complications to be the most common reasons for readmissions. In fact, complications that are Surgical Care Improvement Project target measures, such as surgical site infections and venous thromboembolism, had similar prevalence in both frail and nonfrail patients in our analysis, suggesting the role of national quality improvement measures to minimize these complications.

Certain process measures suggested to decrease readmissions include medication reconciliation, patient education, follow-up telephone calls, and patient-centered discharge instructions.^46^ However, older and frail patients may have significant cognitive, learning, and hearing deficits or functional impairments that may limit the value of such interventions. Readmission reasons, such as pain and dehydration, among frail individuals suggest the inability to manage routine postoperative recovery at home, necessitating readmissions. Minimizing and recognizing postoperative complications, while important, may not be the only solution. In our analysis, more than half of the cohort had no known prespecified NSQIP complications that were determined to be their reason for readmission. Moreover, only 22.8% to 29.3% of the association with frailty was mediated through the occurrence of complications. Even though complications have a significant association with readmissions, they are rare in ambulatory surgery (3.1% in our cohort), are hard to predict, and cannot be evaluated before their occurrence. However, frailty can be measured preoperatively to identify high-risk patients and design care delivery to reduce readmissions. Developing better transitional care models circumventing social, cognitive, and functional barriers that are unique to this high-risk population and engaging caregivers ahead of surgery or discharge to anticipate and manage minor complications at home may help to reduce unplanned readmissions after outpatient surgery.

To improve the surgical care process, it may be prudent to identify frail patients at high risk for postoperative complications and readmissions and develop interventions targeted to the needs of this high-risk subpopulation. Such interventions are complex and costly endeavors; hence, identification of a high-risk subcohort may lead to more efficient interventions, as seen in medical and emergency department populations.^47,48^ Our study suggests that screening for frailty may be an appropriate way to identify the subpopulation for focused interventions, and, as the only frailty tool proven to be feasible for rapid, systemwide, prospective screening of surgical populations, the RAI could be used for real-time identification and triage of patients at high risk for unplanned readmissions.^28^ We also found that the reasons for readmission rarely decreased under one of the predetermined NSQIP categories. More than 70% of the reasons for readmission were either missing or classified as Other. After recategorization of readmission reasons based on work by Merkow et al^3^ (eTable 4 in the Supplement), we were able to better classify and understand the reasons for readmission. However, to better understand and address readmissions, the NSQIP categorizations may benefit from being broadened or adding more categories.

The most recent data from the Centers for Disease Control and Prevention estimate that more than 50 million ambulatory surgery procedures are performed annually, and 33% of these involve patients older than 65 years.^28,49^ Moreover, hospitals are increasingly transitioning to observation status for many diagnoses and procedures, especially in older individuals,^50^ possibly to reduce costs and avoid penalties under the Centers for Medicare & Medicaid Services Hospital Readmission Reduction Program.^51,52^ Currently, only orthopedic procedures are subject to readmission penalties under the Hospital Readmission Reduction Program, and the use of observation and outpatient stays are increasing for these procedures. Inclusion of more surgical procedures in the Hospital Readmission Reduction Program may lead to a further increase in the number of older, frail patients undergoing procedures on an outpatient basis.

### Limitations

Our study has several limitations. Although our readmission rate is commensurate with prior studies, we still may not be capturing all readmissions, especially if patients are admitted to other institutions that were not captured by nurse abstractors and telephone follow-ups. The NSQIP evaluates primarily high-risk surgical procedures such that the outpatient data are limited to hospital-based major outpatient surgical procedures performed under general, spinal, or epidural anesthesia. Therefore, our results cannot be generalized to all outpatient surgical procedures in the United States. However, the association of frailty with increased readmissions in outpatient cases in our study and inpatient surgical procedures as shown by other literature may translate to all ambulatory surgical procedures in future studies. The NSQIP captures readmissions from the date of surgery and not the date of discharge; hence, readmission studies using the NSQIP may experience lead-time bias. However, our study evaluated only outpatient surgical procedures, therefore capturing true 30-day readmission from discharge for most patients. Our study is a retrospective analysis; hence, our model adjusts only for available covariates in the database and may have residual confounding. There is not yet consensus in the literature on the best mediation analytic methods for a binary outcome and binary mediator,^53,54,55^ and a methodological comparison is beyond the scope of this report.

## Conclusions

This study suggests that frail patients have an increased rate of unplanned readmission after ambulatory surgery, and the risk of readmission is partially mediated through the occurrence of postoperative complications. Preoperative identification of these patients may lead to better outcomes and reduced readmissions by improved patient selection and/or improved perioperative care pathways including improved prehabilitation before surgery, frailty-specific anesthetic pathways during surgery, and postoperative follow-up aimed at early identification and outpatient management of complications.

## Notes

Supplement. **eTable 1.** Patient Demographics by Cohort Length of Stay (LOS) = 0 Days vs LOS ≥ 1 Days **eTable 2.** RAI (Risk Analysis Index) Score Components Mapped to NSQIP (National Surgical Quality Improvement Program) Variables **eTable 3.** *CPT* (*Current Procedure Terminology*) Frequency Table by Type of Surgeries Included in the Cohort NSQIP (National Surgical Quality Improvement Program), Stratified by Frailty **eTable 4.** Reasons for Unplanned Readmissions (N = 7,774) by Frailty, Based on NSQIP (National Surgical Quality Improvement Program) Reasons and by Categories Specified by Merkow et al for Patients Undergoing Elective Outpatient Surgery **eFigure.** Cohort Creation **eAppendix.** Methods: Mediation Analysis **eReferences**
