# Supplementary material for: Association of Frailty and Postoperative Complications With Unplanned Readmissions After Elective Outpatient Surgery
Source: JAMA Netw Open. 2019 May 24;2(5):e194330. doi: 10.1001/jamanetworkopen.2019.4330 (PMC6632151; doi:10.1001/jamanetworkopen.2019.4330)
Supplement: Supplement. — eTable 1. Patient Demographics by Cohort Length of Stay (LOS) = 0 Days vs LOS ≥ 1 Days eTable 2. RAI (Risk Analysis Index) Score Components Mapped to NSQIP (National Surgical Quality Improvement Program) Variables eTable 3. CPT (Current Procedure Terminology) Frequency Table by Type of Surgeries Included in the Cohort NSQIP (National Surgical Quality Improvement Program), Stratified by Frailty eTable 4. Reasons for Unplanned Readmissions (N = 7,774) by Frailty, Based on NSQIP (National Surgical Quality Improvement Program) Reasons and by Categories Specified by Merkow et al for Patients Undergoing Elective Outpatient Surgery eFigure. Cohort Creation eAppendix. Methods: Mediation Analysis eReferences [file jamanetwopen-2-e194330-s001.pdf]

Supplementary Online Content

Rothenberg KA, Stern JR, George EL, et al. Association of frailty and postoperative complications with unplanned readmissions after elective outpatient surgery. *JAMA Netw Open.* 2019;2(5):e194330. doi:10.1001/jamanetworkopen.2019.4330

eTable 1. Patient Demographics by Cohort Length of Stay (LOS) = 0 Days vs LOS ≥ 1 Days
eTable 2. RAI (Risk Analysis Index) Score Components Mapped to NSQIP (National Surgical Quality Improvement Program) Variables
eTable 3. *CPT* (*Current Procedure Terminology*) Frequency Table by Type of Surgeries Included in the Cohort NSQIP (National Surgical Quality Improvement Program), Stratified by Frailty
eTable 4. Reasons for Unplanned Readmissions (N=7,774) by Frailty, Based on NSQIP (National Surgical Quality Improvement Program) Reasons and by Categories Specified by Merkow et al for Patients Undergoing Elective Outpatient Surgery
eFigure. Cohort Creation
eAppendix. Methods: Mediation Analysis
eReferences

This supplementary material has been provided by the authors to give readers additional information about their work.

© 2019 Rothenberg KA et al. *JAMA Network Open.*

eTable 1. Patient Demographics by Cohort Length of Stay (LOS) = 0 Days vs LOS ≥ 1 Days

Characteristics	LOS = 0<br>76.8%; N = 321,104	LOS ≥ 1<br>23.2%; N = 96,736	*P* Value
**Mean age, years (SD)**	35.8 (16.4)	38.1 (15.8)	<.001
**Female gender, % [N]**	55.6 [178,610]	71.2 [68,897]	<.001
**Diabetes Mellitus, % [N]**			
Insulin	3.4 [10,909]	4.5 [4,367]	<.001
Non-insulin	7.2 [23,221]	9.2 [8,921]	<.001
**Hypertension, % [N]**	35.3 [113,480]	43.0 [41,634]	<.001
**Congestive Heart Failure, % [N]**	0.2 [602]	0.3 [248]	<.001
**Coronary Artery Disease, % [N]**	0.5 [1,678]	0.7 [628]	<.001
**Disseminated Cancer, % [N]**	0.4 [1,358]	0.8 [803]	<.001
**COPD, % [N]**	2.5 [7,889]	3.4 [3,292]	<.001
**Current Smoker, % [N]**	17.1 [54,904]	17.0 [16,414]	.34
**Preoperative Hematocrit, % [N]**			<.001
Quartile 1: 43.2-60.0	18.3 [58,593]	17.5 [16,904]	
Quartile 2: 40.6-43.1	16.5 [53,099]	20.0 [19,375]	
Quartile 3: 37.9-40.5	17.5 [56,330]	23.8 [23,025]	
Quartile 4: 8.0-37.8	16.1 [51,599]	23.7 [22,929]	
Unknown/Missing	31.6 [101,483]	15.0 [14,503]	
**ASA classification, % [N]**			<.001
I: No Disturbance	17.4 [55,759]	7.6 [7,319]	
II: Mild Disturbance	56.2 [180,522]	54.3 [52,552]	
III: Severe Disturbance	24.4 [78,219]	35.6 [34,419]	
IV/V: Life Threatening/Moribund	1.5 [4,764]	2.3 [2,178]	
Unknown/Missing	0.6 [1,840]	0.3 [268]	
**Steroid use, % [N]**	2.1 [6,697]	2,635 [2.7]	<.001
**Bleeding disorder, % [N]**	1.9 [6,077]	2.6 [2,541]	<.001
**Frail (RAI≥30), % [N]**	2.5 [8,079]	3.1 [3,041]	<.001

Abbreviations: COPD = Chronic Obstructive Pulmonary Disease; ASA = American Society of Anesthesiologists Physical Status Classification; RAI = Risk Analysis Index

© 2019 Rothenberg KA et al. *JAMA Network Open.*

eTable 2. RAI (Risk Analysis Index) Score Components Mapped to NSQIP (National Surgical Quality Improvement Program) Variables

Variable	RAI		NSQIP Variable
Male Sex	3		SEX
Age*Cancer	no cancer	cancer	AGE<br>DISCANCR
Age			
≤19	0	28	
20-24	1	29	
25-29	4	29	
30-34	6	30	
35-39	8	30	
40-44	10	31	
45-49	12	31	
50-54	14	32	
55-59	16	32	
60-64	18	33	
65-69	20	34	
70-74	22	34	
75-79	24	35	
80-84	26	35	
85-89	28	36	
90+	30	36	
Weight Loss	8		WTLOSS
Renal Failure	8		RENAFAIL; DIALYSIS
Congestive Heart Failure	5		HXCHF
Shortness of Breath	3		DYSPNEA
Residence other than Ind. Living	1		TRANST
Activities of Daily Living*Cog	no cognitive	cognitive	FNSTATUS2<br>IMPSENS; COMA; CVA
Totally dependent	14	16	
Partially dependent	7	11	
Independent	0	5	
**Total RAI (range)**	**0**	**81**	

© 2019 Rothenberg KA et al. *JAMA Network Open.*

eTable 3. *CPT* (*Current Procedure Terminology*) Frequency Table by Type of Surgeries Included in the Cohort NSQIP (National Surgical Quality Improvement Program), Stratified by Frailty

**Non-Frail 97.3%; N = 406,720**			**Frail 2.7%; N = 11,120**		
**Surgery Category**	**Percent**	**N**	**Surgery Category**	**Percent**	**N**
**General/GI**	**39.5**	**160,604**	**General/GI**	**38.1**	**4,231**
Laparoscopic Cholecystectomy	7.2	29,395	Inguinal Hernia	10.0	1,113
Inguinal Hernia	5.9	24,043	Laparoscopic Intraperitoneal Cath	3.3	370
Umbilical Hernia	3.6	14,770	Laparoscopic Cholecystectomy	3.0	337
**Skin/Soft Tissue**	**16.8**	**68,782**	Laparoscopic Biopsy	1.8	205
Partial Mastectomy	3.9	15,960	Laparoscopic Inguinal Hernia	1.7	191
Excisional Breast Biopsy	2.5	9,975	**Urology**	**21.5**	**2,392**
Simple Mastectomy	1.4	5,519	Cystoscopic Excision	4.4	488
**Musculoskeletal**	**15.8**	**64,154**	Transurethral Resection	3.3	366
Arthroscopic Knee	2.6	10,575	Laser Prostate Surgery	3.0	338
Arthroscopic Rotator Cuff	1.2	4,965	Cystoscopic Removal Tumor	2.1	231
**Ob/Gyn**	**9.2**	**37,271**	**Skin/Soft Tissue**	**14.7**	**1,638**
Laparoscopic Hysterectomy	1.6	6,601	**Cardiac/Vascular**	**11.8**	**1,316**
Sling	1.4	5,860	Arteriovenous Fistula Banding	1.8	196
**Endocrine**	**5.6**	**22,803**	Cardiac Stent	1.2	135
Thyroidectomy	1.7	6,769	Femoral/Popliteal Angioplasty	1.2	129
Parathyroidectomy	1.5	5,946	Laser Vein Ablation	1.1	119
Thyroid Lobectomy	1.3	5,292	Stab Phlebectomy	1.0	108
**Urology**	**4.9**	**19,782**	**Musculoskeletal**	**6.3**	**703**
**Cardiac/Vascular**	**4.2**	**17,190**	**Endocrine**	**2.5**	**275**
**Neurosurgery**	**3.0**	**12,378**	**Ob/Gyn**	**1.9**	**207**
Lumbar Laminotomy	1.6	6,612	**Chest/Diaphragm**	**1.5**	**169**
**Other**	**0.7**	**2,774**	**Neurosurgery**	**1.5**	**169**
**Chest/Diaphragm**	**0.3**	**1,270**	**Other**	**0.2**	**20**

© 2019 Rothenberg KA et al. *JAMA Network Open.*

eTable 4. Reasons for Unplanned Readmissions (N=7,774) by Frailty, Based on NSQIP (National Surgical Quality Improvement Program) Reasons and by Categories Specified by Merkow et al[1] for Patients Undergoing Elective Outpatient Surgery

**NSQIP Reason, % [N]**	**Non-Frail 91.1%; N=7,081**	**Frail 8.9%; N=693**	***P* Value**
Other	71.1 [5,033]	68.8 [477]	.21
Pneumonia	2.2 [156]	5.3 [37]	<.001
Sepsis	1.8 [130]	4.6 [32]	<.001
Urinary Tract Infection	2.1 [150]	3.6 [25]	.012
Myocardial Infarction	0.8 [57]	2.6 [18]	<.001
Organ/Space SSI	4.9 [344]	2.5 [17]	.004
Superficial Incisional SSI	5.0 [355]	2.2 [15]	.001
Cerebrovascular Accident	0.7 [50]	2.0 [14]	<.001
Pulmonary Embolism	2.9 [202]	1.6 [11]	.05
Septic Shock	0.4 [29]	1.4 [10]	<.001
Acute Renal Failure	0.3 [21]	1.2 [8]	<.001
Deep Incisional SSI	4.1 [293]	1.2 [8]	<.001
Deep Venous Thrombosis Req. Therapy	1.5 [108]	1.0 [7]	.41
Progressive Renal Insufficiency	0.4 [27]	0.7 [5]	.20
Wound Disruption	1.2 [87]	0.7 [5]	.35
Cardiac Arrest Req. CPR	0.07 [5]	0.3 [2]	.12
Bleeding Req. Transfusion	0.3 [24]	0.1 [1]	.72
Graft/Prosthesis/Flap Failure	0.07 [5]	0.1 [1]	.43
On Ventilator > 48 Hours	0.01 [1]	0	>.99
Unplanned Intubation	0.06 [4]	0	>.99
Missing, N	*1,751*	*234*	-
**Reason as classified by Merkow et al[1], % [N]**	**Non-Frail 91.1%; N=7,081**	**Frail 8.9%; N=693**	***P* Value**
Bleeding	11.4 [810]	10.5 [73]	<.001
Ileus or obstruction/Other Gastrointestinal	16.4 [1,164]	10.1 [70]	<.001
Pulmonary	4.4 [310]	9.4 [65]	<.001
Cardiac	3.1 [220]	8.8 [61]	<.001
SSI	18.6 [1,320]	7.5 [52]	.010
Sepsis	2.8 [198]	7.1 [49]	<.001
Other Medical	5.6 [399]	6.9 [48]	<.001
Cancer	5.0 [352]	6.2 [43]	<.001
Acute Kidney Injury/Other Genitourinary	3.7 [265]	5.6 [39]	<.001
Other Surgical	5.0 [356]	4.5 [31]	<.001
Graft/Prosthesis	2.2 [156]	4.5 [31]	<.001
Urinary Tract Infection	2.4 [170]	3.8 [26]	<.001
CNS/Cerebrovascular Accident	1.5 [106]	3.3 [23]	<.001
Deep Venous Thrombosis	4.8 [337]	3.2 [22]	<.001
Dehydration/Nutrition	2.8 [195]	2.6 [18]	<.001
Orthopedic	2.0 [144]	2.3 [16]	<.001
Vascular	1.2 [85]	2.3 [16]	<.001
Pain	7.6 [540]	2.0 [14]	.84
Missing, N	*1,751*	*234*	-

Abbreviations: CPR = Cardiopulmonary Resuscitation; SSI = Surgical Site Infection; CNS = Central Nervous System

© 2019 Rothenberg KA et al. *JAMA Network Open.*

eFigure. Cohort Creation

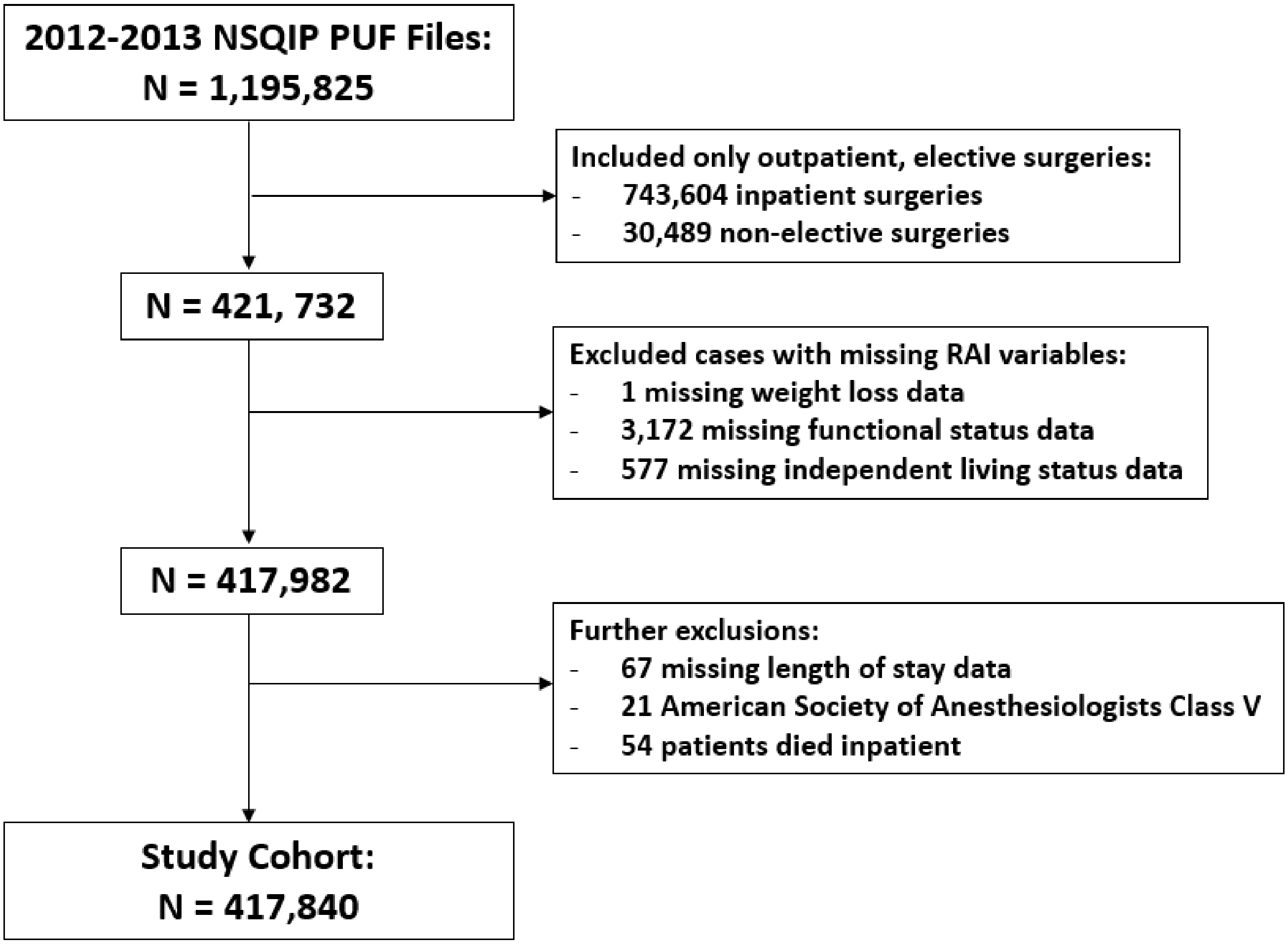

© 2019 Rothenberg KA et al. *JAMA Network Open.*

eAppendix. Methods: Mediation Analysis

We evaluated the effects of frailty on unplanned hospitalizations mediated by post-operative complications. We hypothesized that complications exist in the causal pathway between frailty and readmissions (i.e. frailty leads to higher rates of complications, which causes some unplanned hospitalizations). The first steps of the analyses were to investigate whether mediation is occurring. Second, we aimed to quantify the proportion of the frailty-unplanned hospitalization effect that is mediated through post-operative complications. We utilized logistic regression models to estimate the mediation effects[2,3] and calculated the proportion mediated using the product method with standardized effects.[4]

I. Variables

X = Frailty (exposure)
M = Complications (mediator)
Y = Unplanned Hospitalizations (outcome)

II. Equations

A. Three logistic regression models were calculated for the LOS 0 and LOS 1+ cohorts.

(1) $\log \{p/(1-p)\} = \beta_0 + \beta_1 X + \beta_c$ Covariates
where p is the probability that M=1 (complications occurred)
$\beta_1 = a$

(2) $\log \{p/(1-p)\} = \beta_0 + \beta_1 X + \beta_2 M + \beta_c$ Covariates
where p is the probability that Y=1 (unplanned hospitalizations occurred)
$\beta_2 = b$, $\beta_1 = c'$

(3) $\log \{p/(1-p)\} = \beta_0 + \beta_1 X + \beta_c$ Covariates
where p is the probability that Y=1 (unplanned hospitalizations occurred)
$\beta_1 = c$

B. Sobel method for calculating the standard error of the mediation effect[2]:

(4) m = a * b (mediation effect)

(5) $$\text{Sobel z-score} = \frac{m}{\sqrt{(b^2 se_a{}^2 + a^2 se_b{}^2)}}$$

where se_a is the standard error of a and se_b is the standard error of b (from logistic regression)

The Sobel p-value is drawn from the unit normal distribution, two-tailed z-test: $H_0 = 0$

C. Proportion of Effect Mediated with Standardized Effects[3]

(6) Standardized a = a * SD(X) / $\sqrt{(a^2 * SD(X)^2) + 3.29}$

(7) Standardized b = b * SD(M) / $\sqrt{(c'^2 * Cov(X,M)) + (b^2 * SD(M)^2) + (2bc' * Cov(X,M)) + 3.29}$

(8) Standardized c = c * SD(X) / $\sqrt{(c^2 * SD(X)^2) + 3.29}$

© 2019 Rothenberg KA et al. *JAMA Network Open.*

(9) Proportion Mediated = (Standardized a * Standardized b) / Standardized c

III. Calculations

A. Requirements to claim mediation[3]

1. Exposure (frailty) affects the mediator (complications); i.e. effect a in Figure 1 is significant.

a. LOS 0

$\log\{p/(1-p)\} = -5.40 + 0.58\,X + \beta_c\,\text{Covariates}$ (1)
$a = \beta_1 = 0.58\ (p<0.001)$ (1)

b. LOS 1+

$\log\{p/(1-p)\} = -4.40 + 0.51\,X + \beta_c\,\text{Covariates}$ (1)
$a = \beta_1 = 0.51\ (p<0.001)$ (1)

2. Mediator affects the outcome adjusting for the effect of intervention on outcome (effect b in (2) is significant)

a. LOS 0

$\log\{p/(1-p)\} = -7.05 + 0.74\,X + 3.93\,M + \beta_c\,\text{Covariates}$ (2)
$b = \beta_2 = 3.93\ (p<0.001)$ (2)
$c' = \beta_1 = 0.74\ (p<0.001)$ (2)

b. LOS 1+

$\log\{p/(1-p)\} = -5.77 + 0.49\,X + 3.32\,M + \beta_c\,\text{Covariates}$ (2)
$b = \beta_2 = 3.32\ (p<0.001)$ (2)
$c' = \beta_1 = 0.49\ (p<0.001)$ (2)

3. The mediation effect is significant (for example, $a \times b \neq 0$)

a. LOS 0

$a = 0.58,\ b = 3.93,\ se_a = 0.05,\ se_b = 0.03$ (1, 2)
$m = a * b = 0.58 * 3.93 = 2.28$ (4)
$\text{Sobel } z = \frac{2.28}{\sqrt{((3.93^2\ x\ 0.05^2) + ((0.58^2\ x\ 0.03^2)}} = 11.6$ (5)
Sobel p <0.001

b. LOS 1+

$a = 0.51,\ b = 3.32,\ se_a = 0.07,\ se_b = 0.04$ (1, 2)
$m = a * b = 0.51 * 3.32 = 1.69$ (4)
$\text{Sobel } z = \frac{1.69}{\sqrt{((3.32^2\ x\ 0.07^2) + ((0.51^2\ x\ 0.04^2)}} = 7.26$ (5)
Sobel p <0.001

© 2019 Rothenberg KA et al. *JAMA Network Open.*

B. Proportion mediated

a. LOS 0

$\log\{p/(1-p)\} = -6.7 + 0.82\,X + \beta_c$ Covariates (3)

$c = \beta_1 = 0.82$ ($p<0.001$) (3)

Standardized a = $0.58 * 0.1566 / \sqrt{(0.58^2 * 0.1566^2) + 3.29} = 0.050$ (6)

Standardized b = (7)

$3.93 * 0.1579 / \sqrt{(0.74^2 * 0.0011) + (3.93^2 * 0.1579^2) + (2 * 3.93 * 0.74 * 0.0011) + 3.29} = 0.322$ (7)

Standardized c = $0.82 * 0.1566 / \sqrt{(0.82^2 * 0.1566^2) + 3.29} = 0.071$ (8)

Proportion Mediated = (0.050 * 0.322) / 0.071 = 0.228 = 22.8% (9)

b. LOS 1+

$\log\{p/(1-p)\} = -5.5 + 0.64\,X + \beta_c$ Covariates (3)

$c = \beta_1 = 0.64$ ($p<0.001$) (3)

Standardized a = $0.51 * 0.1745 / \sqrt{(0.51^2 * 0.1745^2) + 3.29} = 0.049$ (6)

Standardized b = (7)

$3.32 * 0.2128 / \sqrt{(0.49^2 * 0.0016) + (3.32^2 * 0.2128^2) + (2 * 3.32 * 0.49 * 0.0016) + 3.29} = 0.362$ (7)

Standardized c = $0.64 * 0.1745 / \sqrt{(0.64^2 * 0.1745^2) + 3.29} = 0.061$ (8)

Proportion Mediated = (0.049 * 0.362) / 0.061 = 0.293 = 29.3% (9)

© 2019 Rothenberg KA et al. *JAMA Network Open.*

eReferences.

1. Merkow RP, Ju MH, Chung JW, et al. Underlying reasons associated with hospital readmission following surgery in the United States. *JAMA*. 2015;313(5):483-495. doi:10.1001/jama.2014.18614

2. Sobel ME. Direct and indirect effects in linear structural equation models. *Sociol Methods Res*. 1987;16:155-176. doi:10.1177/0049124187016001006

3. Mascha EJ, Dalton JE, Kurz A, Saager L. Statistical grand rounds: understanding the mechanism: mediation analysis in randomized and nonrandomized studies. *Anesth Analg*. 2013;117(4):980-994. Medline:24023021 doi:10.1213/ANE.0b013e3182a44cb9

4. Baron RM, Kenny DA. The moderator-mediator variable distinction in social psychological research: conceptual, strategic, and statistical considerations. *J Pers Soc Psychol*. 1986;51(6):1173-1182. Medline:3806354 doi:10.1037/0022-3514.51.6.1173

© 2019 Rothenberg KA et al. *JAMA Network Open.*
